# Supplementary material for: From sequence to enzyme mechanism using multi-label machine learning
Source: BMC Bioinformatics. 2014 May 19;15:150. doi: 10.1186/1471-2105-15-150 (PMC4229970; doi:10.1186/1471-2105-15-150)
Supplement: Additional file 2 — Java code of ml2db. Additional file ml2db_code.tar.gz contains the Java source code to run the multi-label machine learning experiments and save the results to database. The code’s Javadoc is included. [file 1471-2105-15-150-S2.zip › additional file 2/ml2db/ml2db/doc/mulan/classifier/meta/RAkEL.html]

RAkEL


JavaScript is disabled on your browser.


- Overview
- Package
- Class
- Use
- Tree
- Deprecated
- Index
- Help

- Prev Class
- Next Class

- Frames
- No Frames

- All Classes

- Summary:
- Nested |
- Field |
- Constr |
- Method

- Detail:
- Field |
- Constr |
- Method


mulan.classifier.meta

## Class RAkEL

- java.lang.Object
- - mulan.classifier.MultiLabelLearnerBase
  - - mulan.classifier.meta.MultiLabelMetaLearner
    - - mulan.classifier.meta.RAkEL

- All Implemented Interfaces:
  :   java.io.Serializable, MultiLabelLearner, weka.core.TechnicalInformationHandler

  ---

    

  ```
  public class RAkEL
  extends MultiLabelMetaLearner
  ```

  Class implementing a generalized version of the RAkEL (RAndom k-labELsets) algorithm. For more information, see

  Version:
  :   2012.1.27

  Author:
  :   Grigorios Tsoumakas

  See Also:
  :   Serialized Form

- - ### Constructor Summary

    Constructors

    Constructor and Description

    `RAkEL()`

    Default constructor

    `RAkEL(MultiLabelLearner baseLearner)`

    Creates an instance based on a given multi-label learner

    `RAkEL(MultiLabelLearner baseLearner,
    int models,
    int subset)`

    Creates an instance given a specific multi-label learner, number of
    models and size of subsets

    `RAkEL(MultiLabelLearner baseLearner,
    int models,
    int subset,
    double threshold)`

    Creates an instance given a specific multi-label learner, number of
    models, size of subsets and threshold
  - ### Method Summary

    Methods

    | Modifier and Type | Method and Description |
    | --- | --- |
    | `static int` | `binomial(int n, int m)` The binomial function |
    | `int` | `getNumModels()` Returns the number of models |
    | `int` | `getSizeOfSubset()` Returns the size of the subsets |
    | `weka.core.TechnicalInformation` | `getTechnicalInformation()` Returns an instance of a TechnicalInformation object, containing detailed information about the technical background of this class, e.g., paper reference or book this class is based on. |
    | `java.lang.String` | `globalInfo()` Returns a string describing classifier |
    | `void` | `setNumModels(int models)` Sets the number of models |
    | `void` | `setSeed(int x)` Sets the seed for random number generation |
    | `void` | `setSizeOfSubset(int size)` Sets the size of the subsets |

    - ### Methods inherited from class mulan.classifier.meta.MultiLabelMetaLearner

      `getBaseLearner`
    - ### Methods inherited from class mulan.classifier.MultiLabelLearnerBase

      `build, getDebug, isUpdatable, makeCopy, makePrediction, setDebug`
    - ### Methods inherited from class java.lang.Object

      `equals, getClass, hashCode, notify, notifyAll, toString, wait, wait, wait`

- - ### Constructor Detail


    - #### RAkEL

      ```
      public RAkEL()
      ```

      Default constructor


    - #### RAkEL

      ```
      public RAkEL(MultiLabelLearner baseLearner)
      ```

      Creates an instance based on a given multi-label learner

      Parameters:
      :   `baseLearner` - the multi-label learner


    - #### RAkEL

      ```
      public RAkEL(MultiLabelLearner baseLearner,
           int models,
           int subset)
      ```

      Creates an instance given a specific multi-label learner, number of
      models and size of subsets

      Parameters:
      :   `baseLearner` - a multi-label learner
      :   `models` - a number of models
      :   `subset` - a size of subsets


    - #### RAkEL

      ```
      public RAkEL(MultiLabelLearner baseLearner,
           int models,
           int subset,
           double threshold)
      ```

      Creates an instance given a specific multi-label learner, number of
      models, size of subsets and threshold

      Parameters:
      :   `baseLearner` - a multi-label learner
      :   `models` - a number of models
      :   `subset` - a size of subsets
      :   `threshold` - a threshold
  - ### Method Detail


    - #### getTechnicalInformation

      ```
      public weka.core.TechnicalInformation getTechnicalInformation()
      ```

      Returns an instance of a TechnicalInformation object, containing detailed
      information about the technical background of this class, e.g., paper
      reference or book this class is based on.

      **Specified by:**
      :   `getTechnicalInformation` in interface `weka.core.TechnicalInformationHandler`

      **Specified by:**
      :   `getTechnicalInformation` in class `MultiLabelLearnerBase`

      Returns:
      :   the technical information about this class


    - #### setSeed

      ```
      public void setSeed(int x)
      ```

      Sets the seed for random number generation

      Parameters:
      :   `x` - the seed


    - #### setSizeOfSubset

      ```
      public void setSizeOfSubset(int size)
      ```

      Sets the size of the subsets

      Parameters:
      :   `size` - the size of the subsets


    - #### getSizeOfSubset

      ```
      public int getSizeOfSubset()
      ```

      Returns the size of the subsets

      Returns:
      :   the size of the subsets


    - #### setNumModels

      ```
      public void setNumModels(int models)
      ```

      Sets the number of models

      Parameters:
      :   `models` - number of models


    - #### getNumModels

      ```
      public int getNumModels()
      ```

      Returns the number of models

      Returns:
      :   number of models


    - #### binomial

      ```
      public static int binomial(int n,
                 int m)
      ```

      The binomial function

      Parameters:
      :   `n` -
      :   `m` -

      Returns:


    - #### globalInfo

      ```
      public java.lang.String globalInfo()
      ```

      Returns a string describing classifier

      **Specified by:**
      :   `globalInfo` in class `MultiLabelLearnerBase`

      Returns:
      :   a description suitable for displaying


- Overview
- Package
- Class
- Use
- Tree
- Deprecated
- Index
- Help

- Prev Class
- Next Class

- Frames
- No Frames

- All Classes

- Summary:
- Nested |
- Field |
- Constr |
- Method

- Detail:
- Field |
- Constr |
- Method
